# Psychometric properties of the Iranian version of self-care ability scale for the elderly

**DOI:** 10.1186/s12877-020-01775-6

**Published:** 2020-09-22

**Authors:** Mehrdad Amir-Behghadami, Jafar Sadegh Tabrizi, Mohammad Saadati, Masoumeh Gholizadeh

**Affiliations:** 1 Student Research Committee (SRC), Tabriz University of Medical Sciences, Tabriz, Iran; 2 Iranian Center of Excellence in Health Management (IceHM), Department of Health Service Management, School of Management and Medical Informatics, Tabriz University of Medical Sciences, University Rd, Golbad, EAZN, Tabriz, 5165665811 Iran; 3 Tabriz Health Service Management Research Centre, Tabriz University of Medical Sciences, Tabriz, Iran; 4 Road Traffic Injury Research Center, Tabriz University of Medical Sciences, Tabriz, Iran

**Keywords:** Elderly, Self-care ability, Instrument, Psychometric, Validity, Reliability

## Abstract

**Background:**

Measuring self-care ability in elderly people needs specific instruments. The Self-care Ability Scale for Elderly (SASE) is one of the common instruments used for assessing self-care ability. The aim of this study was to assess the psychometric properties of the SASE among Iranian elderly population.

**Methods:**

This cross-cultural adaptation study was carried out at Shahid Chamran and Shadpour Health Complex in Tabriz, Iran. The forward-backward procedure was applied to translate the SASE from English into Persian. Then, it was completed to 220 elderly people. A systematic random sampling method was used for sampling. Content validity was calculated through modified Kappa coefficient (modified CVI) based on clarity and relevance criteria. Reliability was measured by internal consistency and test-retest analysis. The construct validity also was assessed using Exploratory Factor Analysis (EFA). All the statistical analyses were performed using SPSS 21 statistical software package.

**Results:**

The mean of self-care ability was 61.14 ± 21.08. The CVI and modified kappa were 0.91 and 0.92 for relevance and clarity, respectively. The Cronbach’s alpha coefficient was 0.73 and Intra-class correlation coefficient was 0.97. The results of EFA revealed a three-factor solution (‘ability to take care of personal responsibility’, ‘ability to take care for the goals’, and ‘ability to take care of the health’) that jointly explained for 64.61% of the total variance.

**Conclusion:**

Results of the study showed that the Iranian version of the SASE has good psychometric properties and can be used in assessing the self-care ability of elderly people.

## Background

Agedness is one of the emerging phenomena in the world of health and one of the natural stages of human life from birth to aging, which includes the dynamics of biological processes, perception, development, and puberty, and leads to physiological, psychological and social changes [1]. During this period, the elderly lose their independence due to physical and mental disabilities [2].

Many nations have identified a rapid growth in the population of elderly people as the phenomenon of the twenty-first century [3]. The population of the elderly is estimated to be above 605 million and it is estimated that by the year 2050 this number will reach two billion people [4]; which also is more than children population growth [5]. Statistical indicators show that the process of aging people in our country is also expanding. According to the United Nations Information Center in Tehran, the number of elderly people in Iran was 4,562,000, which means 6% of the total population of Iran is over 60 years old [1]. Aging occurs in all main regions of the world; on the whole, proportion of the people over 60 years old was 9% in 1994 but increased up to 12% in 2014. And, it is expected to hit 21% in 2050 [6].

The growing ageing population creates demands on the healthcare system to strengthen the older person’s self-care ability and to promote healthy ageing [7]. Although aging is often associated with a reduction in physiological, cognitive and functional abilities, healthy aging does not mean the absence of restrictions but a level of health and compliance with the aging process that is acceptable to people. The literature emphasizes factors that may be positively attributed to healthy aging. Farquhar mentioned the dimensions of quality of life that are often mentioned by older people: family, social relationships, health (physical), mobility / ability, activities, happiness, youthfulness, and home environment [8]. Factors relevant to the elderly health and self-care can be used for regular assessment to support and promote healthy ageing [7].

Self-care ability is a determinant factor in managing the daily lives of the elderly [9]. Self-care is taken as a personal care, which older people do for themselves to maintain, restore or promote their health [10]. Self-care refers to the individual’s ability to perform self-care or self-management activities that has achieved the balance between their abilities and the existing needs for self-care and is influenced by factors such as age, level of development, life experiences, and cultural context and health conditions. Self-care ability is different in different stages of development, as well as in acute or chronic conditions of health [11]. However, high self-care ability can also be affected by health (physical and mental), good habits (for example, a healthy lifestyle such as being active, having a satisfactory social relationship), self-esteem, and self-care ability [12]. It is also a major source of health and well-being for the elderly, especially when they live in the home [13]. Salehi et al. reported that indolence is the most significant obstacle for not being engaged in physical activities [14]. Similarly, they reported in another study that some reasons for not being active are fear of falling, lack of motivation, unsafe physical environment, extensive tiredness, illness, and not having a companion to do physical activities [15, 16]. A study in Iran has reported that the demand for health care in elderly people is 3 times more than the non-elderly population, as the age of the elderly increases, this demand also increases [17]. However, health centers do not respond to the increasing demand of this population. Therefore, increasing the responsibility for self-care allows the elderly to be less dependent on them and improve their quality of life [1].

Rechel et al. show that health systems can support elderly people by encouraging them to have self-care [18]. Self-care increases self-promoting behaviors, or the ability of individuals to self-manage chronic diseases [19]. Therefore, the self-care ability of elderly people should be turned into international issues and be important for health professionals around the world. Using instruments to assess self-care is a starting point for encouraging older people to take care of themselves [20].

Health care providers need to know the self-care ability of individuals for identifying people at risk and developing their self-care programs at the individual and social level [21]. Therefore, it is essential that the reliability and validity instruments be designed to measure self-care [22]. In addition, using of a viable and reliable instrument, which measures self-care, can stimulate further research on health promotion and self-management of chronic diseases. Such studies can help people in their care activities to manage their chronic illnesses and preventing it [19].

The original Swedish version of the SASE has been tested, and there is evidence to show its validity and reliability [23, 24]. In addition, it is short (17 questions) and easy to complete. Considering the growth of the aging population in Iran and the need to provide health, medical and social services in accordance with their needs; measuring self-care ability in the elderly is a necessary issue. There are few instruments specifically for those with heart failure [25] and diabetic patients [26] not for general elderly population. Therefore, the purpose of study was to assess the psychometric properties of the Iranian version of the SASE in order to provide a standard, valid, and reliable instrument for measuring the elderly’s self-care ability.

## Methods

### Study design and sample

In recent years, the increase in the number of multicultural studies has urged the need to adapt scales to be used in other languages. Hence, depending on different cultures, the scales should be culturally modified and adapted. Regarding this point, cross-cultural adaptation must be applied as the study design. This consists of translation, adaptation, calculation of validity, and reliability. The present study was also a cross-cultural adaptation conducted in Shahid Chamran and Shadpour health complexes affiliated to Tabriz University of Medical Sciences in Tabriz, Iran 2015. Health complexes were launched after implementation of the health system reform plan in Iran, which assumed to provide integrated care in a defined area under district health center policies and regulations. Each health complex consists of 4–5 health centers that cover 10,000 to 30,000 people. Health centers generally provide preventive services and, if necessary, refer individuals to more specialized services to the health complex clinics.

### Sampling and sample size calculation

Sample framework for selecting people was based on the registered records health. As the samples of this study, 220 older people who attend these complexes were recruited. The sample size of 220 people was calculated using G-Power software. Sample allocation to each complex was done in the same way. An a priori G-Power analysis was performed. Thus, considering independent samples t-test (two tailed) with 99% power and alpha = 0.01 significance level, we estimated an overall sample size of 220 older people for detecting differences between two independent means with effect sizes (d = 0.66). The participants were selected using a systematic random sampling method according to the following inclusion criteria: age over 60 years; ability to communicate; having a stable condition; and willingness to take part in this study. People, who were not permanent residents of Shahid Chamran and Zafaraniyeh or who suffered from acute disease, were excluded from this study.

### Instrument

The SASE, as a self-reporting instrument, measures self-care ability among older people [25]. In fact, three versions were available in Chinese, Italian and Swedish. This instrument consists of two parts: the first part, the demographic characteristics of the elderly, and the second part consists of 17-item Likert scale that highlights areas of particular importance to self-care for the elderly, i.e. daily life activities, welfare, power, desires, determination, loneliness and wear clothes. The accountability scale of each item is between 1 “totally disagree” to 5 “totally agree”. A higher overall score indicates a higher self-care ability. The construct of self-care ability is considered as a necessary condition for self-care, and when this ability is exercised, self-care actions are achieved. The internal structure of SASE includes “ability for care of repertoire”, “ability for care of goal”, and “ability for care of well-being” [23, 24] .

### Translation of the instrument

The permission to translate the English version of the SASE was obtained from the main author (Söderhamn). Then, the forward-backward translation and cultural adaptation was performed at several steps. A professional translator translated the instrument into Persian and another professional translator translated the Iranian version back into English. Both translators performed the translation process separately. After the completion of translation, an expert panel consisting of two elderly specialists and three elderly nursing compared the original English version with back translation and following cultural and linguistic adaptation, pre-final Iranian version of the SASE was prepared. Then, this version was examined among ten older people and modifications were done based on their feedback. At last, it was developed and used in this study for further psychometric assessment.

### Statistical analysis

Descriptive statistics were used to describe the characteristics of the sample and several statistical analyses were applied to assess the psychometric properties of the Iranian version of SASE.

#### Content validity

The content validity was assessed through modified KAPPA by means of qualitative and quantitative approaches [27]. In this regard, the Iranian version of the SASE was sent to 10 specialists with sufficient clinical experience and theoretical knowledge about nursing and self-care. The fields of the experts were health education and promotion, geriatric health, community health, psychology, nursing, and epidemiology. In the qualitative approach, experts were requested to provide their own ideas for improving its quality. And in the quantitative approach, in order to calculate the CVI and modified KAPPA coefficient basis on relevance and clarity, each item was ranked on a four-point Likert scale based on views of the experts [27, 28].

#### Reliability

The reliability was assessed using internal consistency and the test–retest reliability method. The internal consistency was measured by the coefficient Cronbach’s alpha, which varies from 0 to 1, and values equal to or > 0.70 for a scale show a satisfactory internal consistency [29]. In order to estimate the test-retest reliability, the intra-class correlation coefficients (ICCs) during a two-week interval were calculated among 30 older people for total scale and each subscale. The following categories were chosen for the interpretation of agreement levels: 00–0.2 as small, 0.21–0.40 as fair, 0.41–0.60 as moderate, 0.61–0.80 as remarkable and 0.81–1 as approximately perfect [30].

#### Exploratory factor analysis

Prior to EFA, Kaiser-Meyer-Olkin (KMO) was calculated to ensure that the sample size was adequate. KMO shows values between 0 and 0.49 as unacceptable, 0.5–0.7 as mediate; 0.7–0.8 as good, 0.8–0.9 as great, and > 0.9 as excellent [31]. EFA was performed using the principal component analysis with varimax solution [32]. Since we could not find a clear relationship between the items in the questions and the construct to be measured, we needed an exploratory factor analysis. In other words, we were not sure which items measured which structures. Therefore, because of this suspicion, we used exploratory factor analysis [33]. To select the factors, an eigenvalue greater than 1 and a factor loading equal to or greater than 0.4 were used. All the statistical analyses were performed using SPSS 21 statistical software package.

## Results

### The sample

The mean age of respondents was 68.65 ± 7.17 and 52.3% of them were male. In addition, 52.3% of the elderly were married, 51.8% of them were illiterate, 39.1% of them had social security insurance, and 48.2% of them had complementary insurance. Additionally, 56.4% of them were living with their spouses and income source of 41.8 of them was pension. The mean of self-care ability in urban living the elder people of Tabriz was 61.14 ± 21.08 (Table 1).

**Table 1:** Mean, SD for self-care ability and its dimensions

Self-care ability and its dimensions				
Statistical indicators	Self-care ability	Ability to take care of personal responsibility	Ability to take care of goals	Ability to take care of health
Mean	61.14	59.60	53.86	54.60
SD	21.08	18.03	18.68	27.85

### Validity

In the qualitative content validity, the Iranian version of the SASE was improved by the experts’ comments. The result of quantitative content validity indicated that the CVI and modified kappa were 0.91 and 0.91 for relevance and 0.92 and 0.92 for clarity, respectively. The CVI and modified Kappa have been illustrated for each item (Table 2).

**Table 2:** The CVI and modified Kappa for each item

Items	Relevance		Clarity	
	CVI	Modified Kappa (Modified CVI)	CVI	Modified Kappa (Modified CVI)
1	1	1	1	1
2	0.9	0.89	1	1
3	0.9	0.89	0.9	0.89
4	0.88	0.88	1	1
5	0.9	0.89	0.9	0.89
6	0.88	0.88	0.88	0.88
7	1	1	1	1
8	0.9	0.89	0.8	0.79
9	0.88	0.88	0.88	0.88
10	1	1	1	1
11	1	1	1	1
12	0.88	0.88	0.88	0.88
13	0.8	0.79	0.9	0.89
14	0.88	0.88	0.88	0.88
15	1	1	0.88	0.88
16	0.8	0.79	0.9	0.89
17	0.9	0.89	0.9	0.89

^a^ *CVI* Content Validity Index

### Reliability

The Cronbach’s alpha coefficient for overall scale, subscale 1, subscale 2, and subscale 3 was 0.73, 0.7, 0.71, and 0.75, respectively. Test–retest reliability for overall scale, subscale 1, subscale 2, and subscale 3 (assessed by ICC) was 0.96, 0.92, 0.85, and 0.97, respectively.

### Exploratory factor analysis

The KMO indicated that the sample size was adequate for factor analysis (KMO index = .757). Three factors were identified with an eigenvalue greater than one that jointly accounted for 64.61% of variance observed. The factor loadings were as follows:

- Factor 1 (Ability to take care of personal responsibility) including 8 items (item 1, 2, 3, 4, 5, 13, 14, and 17).
- Factor 2 (Ability to take care of goals) including 7 items (item 6, 7, 8, 9, 11, 12, and 16).
- Factor 3 (Ability to take care of health) including 2 items (item 10 and 15). (Table 3)

**Table 3:** The results obtained from EFA of the Iranian version of SASE

Subscales	Items	Factors		
		F1	F2	F3
**Ability to take care of personal responsibility**	1	0.723		
	2	0.745		
	3	0.612		
	4	0.727		
	5	0.883		
	13	0.625		
	17	0.514		
**Ability to take care of goals**	6		0.726	
	7		0.645	
	8		0.633	
	9		0.653	
	11		0.814	
	12		0.749	
	14		0.589	
	16		0.453	
**Ability to take care of health**	10			0.825
	15			0.544
Eigen Value	–	5.73	2.86	2.37
Variance	–	33.76%	16.90%	13.96%

^a^ *EFA* Exploratory Factor Analysis

## Discussion

The SASE has been assessed and used in some studies among older people [23, 24]. Measuring self-care ability in the elderly people plays an important role in improving their quality of life. In general, the results of the current study demonstrated that Iranian version of the SASE had a valid and reliable instrument for assessing self-care ability in the Iranian older population.

Content validity is an essential component of psychometric properties, which must be done independent of the translation phase. During the qualitative content validity, only a few points with trivial ambiguity were determined by the experts and were modified accordingly [1]. In the quantitative content validity, the CVI and modified kappa were also assessed and the calculated values were at a high level. Therefore, the Iranian version of the SASE indicated good content validity. In studies by Söderhamn et al. (1996) and Tomstad et al. (2013), the content validity was not calculated [22, 23].

The internal consistency of scale was satisfactory (alpha 0.73), indicating that the instrument is homogeneous. The obtained alpha in this study is higher than the reported alpha in the Swedish study (alpha 0.68) [9]; however, it is less than the reported alpha in the Norwegian and Swedish studies, respectively (alphas 0. 88 and 0.85) [22, 23]. In general, a satisfactory level of internal consistency was considered a Cronbach’s α ≥ 0.7 for scale and all of the subscales. The test-retest reliability of the SASE with a two-week interval was found to have almost perfect stability. Using the intra-class correlation coefficients test, all subscales reported a high correlation (≥ 0.8). In a study by Gao et al. (2017), the test-retest reliability was (≥ 0.8) similar to this study [20]; however, it had not been measured in studies by Söderhamn et al. (1996) and Tomstad et al. (2013) [22, 23].

The results KMO confirmed that the sample size was adequate for factor analysis. The EFA showed a three-factor solution to the Iranian version of the SASE, which was similar to the study conducted by Olle Söderhamn (2001) and the study done by Gao et al. (2017), a three-factor solution including components such as repertoire (factor 1), goal (factor 2), and environment (factor 3). However, the three factors EFA found in this study differ from the five factors EFA reported in study Söderhamn et al. [20, 23]. This five-factor solution accounted for 70.2% of the variance. Factor 1 represented the ability of care (responsibility and environment). Factor 2 represented the ability to care (purpose and responsibility). Factor 3 represented the goals and ability to take care of responsibility. Factor 4 represented the ability to take care (responsibility), and factor 5 represented the ability to take care of the goals. Study finding indicated a three-factor solution that totally explained 64.61% of the variance.

## Conclusion

This study showed that the Iranian version of the SASE has good psychometric properties to be used. Therefore, it will help nurses and health care providers in different health care centers such as hospitals, primary health-care centers, and rehabilitation centers to evaluate elderlies’ self-care ability. This study had some limitations and strengths. Firstly, a major problem with the translation is that the English version was used for translation. The most accurate method was to use Swedish to Persian translation. However, professional translators translated the Swedish version of the SASE according to the recommended procedure in English (Brislin 1970). Moreover, the rigorous translation methods used in the present study were used to obtain a good translation of the English version of SASE to Persian. Secondly, it is likely that study results may not be completely generalizable to all the elderly, since it was carried out on older people referring to health complexes. Hence, other scholars should assess the validity and reliability of the instrument and then use it in accordance with their needs. Finally, we have not tested concurrent validity. Therefore, it is suggested that further research be conducted to include other validated questionnaire such as Physical Activity Scale for Elderly (PASE) [15] to provide stronger support for concurrent validity. Independent expert panels for translating and evaluating content validity of scale are strength of the study.

## Data Availability

The datasets used and/or analyzed during the current study are available from the corresponding author on reasonable request.

## Abbreviations

SASE: Self-care Ability Scale for Elderly
EFA: Exploratory Factor Analysis
ICC: Intra-class Correlation Coefficients
KMO: Kaiser-Meyer-Olkin
CHC: Community Health Center
PASE: Physical Activity Scale for Elderly
